# The use of intraoperative cell salvage in total hip arthroplasty with subtrochanteric shortening osteotomy for the treatment of high hip dislocation: a retrospective cohort study

**DOI:** 10.1186/s12891-023-06427-5

**Published:** 2023-04-22

**Authors:** Enze Zhao, Xiaoyan Zhu, Kai Zhou, Zunhan Liu, Hanpeng Lu, Jiali Chen, Zongke Zhou

**Affiliations:** 1 Department of Orthopedic Surgery, West China Medical School, West China Hospital, Sichuan University, Chengdu, Sichuan Province 610041 People’s Republic of China; 2 West China School of Nursing, Department of Orthopedics, West China Hospital, Sichuan University, Chengdu, Sichuan Province 610041 People’s Republic of China; 3 Department of Sports Medicine Center, State Key Laboratory of Trauma, Burn and Combined Injury, the First Affiliated Hospital of the Army Military Medical University, Chongqing, China; 4 Department of Orthopedics, West China Hospital, Sichuan University, #37 Guoxue Road, Chengdu, 610041 People’s Republic of China

**Keywords:** Cell salvage, Total hip arthroplasty, Subtrochanteric shortening osteotomy, Allogeneic blood transfusion

## Abstract

**Background:**

Intraoperative cell salvage (ICS) is an important component of blood management in patients undergoing orthopedic surgery. However, the role of ICS is less well defined in total hip arthroplasty (THA) with subtrochanteric shortening osteotomy (SSO) which is a common surgical technique to manage high hip dislocation. This study aimed to determine the effect of ICS during THA with SSO and to identify factors associated with the ability to salvage sufficient collection for reinfusion in patients with high hip dislocation.

**Methods:**

We identified 178 patients who underwent THA with SSO for high hip dislocation between November 2010 and April 2021. The consecutive cohort was analyzed by logistic regression to determine the effect of ICS on postoperative allogeneic blood transfusion (ABT) and to explore the associations between patient demographics, clinical and radiographic characteristics, preoperative laboratory examination, and surgical variables with the ability to generate adequate blood salvage to reinfuse.

**Results:**

In the consecutive cohort of 178 patients, cell salvage was reinfused in 107 patients (60.1%) and postoperative allogeneic red blood cell (RBC) transfusion within 3 days of implantation was administrated in 40 patients (22.5%). In multivariate analysis, the reinfusion of ICS (OR (95%CI) 0.17 (0.07–0.47)), center of rotation (COR) height ≥ 60 mm (OR (95%CI) 3.30 (1.21–9.01)), the length of SSO ≥ 30 mm (OR (95%CI) 2.75 (1.05–7.22)) and the use of drainage (OR (95%CI) 2.28 (1.04–5.03)) were identified as independent factors of postoperative allogeneic RBC transfusion. In addition, the following variables were identified as independent factors associated with the ability to generate sufficient blood salvage volume for reinfusion: COR height ≥ 60 mm (OR (95%CI) 3.47 (1.58–7.61)), limb-length discrepancy (LLD) ≥ 25 mm (OR (95%CI) 2.55 (1.15–5.65)) and length of SSO ≥ 30 mm (OR (95%CI) 2.75 (1.33–5.69)).

**Conclusions:**

ICS was efficacious in reducing the exposure rate of postoperative RBC transfusion for high hip dislocation during THA with SSO. In addition, patients with greater COR height, larger LLD, and longer length of SSO were predisposed to generate sufficient collection for reinfusion in THA with SSO.

## Introduction

High hip dislocation (Crowe type III and IV) secondary to developmental dysplasia of the hip (DDH) is one of the most challenging types of hip deformity to reconstruct. Anatomic reconstruction of the hip rotation center is considered the gold standard treatment for high hip dislocation [1]. Normally restoration of the hip rotation center into the anatomic acetabulum requires total hip arthroplasty (THA) combined with subtrochanteric shortening osteotomy (SSO) to decrease the risk for nerve stretching [2–4]. However, THA combined with SSO is a demanding surgical technique with substantial intraoperative blood loss and high rate of allogeneic red blood cell (RBC) transfusion [2–4]. It was reported that perioperative blood loss of THA with SSO could reach over 1000 ml with an average of 2 units of allogeneic RBC transfusion [5]. Allogeneic blood transfusion has been reported to be an independent risk factor associated with mortality and complication such as surgical-site infection and venous thrombosis [6, 7]. Effective methods are used to reduce exposure to allogeneic transfusion including hypotensive anesthesia, antifibrinolytic therapy, and cell salvage system [8–10].

Intraoperative cell salvage (ICS) provides an efficacious approach to decrease the exposure of allogeneic blood transfusion (ABT) in THA [10]. However, there are no published data regarding the role of ICS in THA combined with SSO. In addition, a recent meta-analysis has reported that ICS reduced neither the rate nor the volume of allogeneic RBC transfusion in THA [11]. Therefore, the exact effect of ICS should be defined. Furthermore, resources and effectiveness also need to be considered in the use of ICS. Adequate blood for processing and reinfusion was collected intraoperatively in only part of the patients due to the administration of antifibrinolytic therapy and intraoperative blood pressure control [8, 9]. A prior study reported that sufficient blood for reinfusion was salvaged in only half of all patients during aseptic elective hip revision arthroplasty [12]. Therefore, it is critical to target patients who are most likely to benefit from ICS for the purpose of management-related decision-making.

The aims of the study were (1) to determine whether the use of ICS could reduce the rate of postoperative allogeneic RBC transfusion in patients who received THA with SSO and (2) to identify potential preoperative factors associated with the ability to salvage sufficient blood to permit processing and reinfusion during THA combined with SSO.

## Methods

### Patients

This study has been approved by the Clinical Trials and Biomedical Ethics Committee of West China Hospital, Sichuan University, and registered with the Chinese Clinical Trial Registry (ID: ChiCTR2200064064). Between November 2010 and April 2021, data on consecutive patients who were diagnosed with Type-III or IV DDH based on Crowe classification and underwent cementless THA combined with SSO in our institution were retrospectively collected in our institution. The exclusion criteria comprised patients who (1) underwent THA combined with SSO due to high hip dislocation secondary to pyogenic arthritis; (2) underwent operative intervention for the purpose of treating DDH before THA combined with SSO; (3) had dysfunction of thromboembolism or underwent preoperative anticoagulation therapy (excluding aspirin); (4) received ABT intraoperatively or refused ABT perioperatively; (5) did not follow the routine practices of tranexamic acid and hypotensive anesthesia in our institution: tranexamic acid was given intravenously 2 g 5 min before incision, and administrated 1 g 3 and 6 h after the surgery; maintaining the systolic blood pressure at 90–100 mmHg during the operation.

### Surgery and management

We used cementless femoral and acetabular prostheses for all patients. All surgeries were designed with transparencies as Krych described [13]. If the affected leg would be lengthened by > 3–4 cm in preoperative measurement, the SSO was administrated to avoid nerve stretching. All patients received general anesthesia, posterolateral approach, and transverse SSO as Wang et al. described [14]. Normally, a transverse femoral osteotomy was conducted approximately 8–10 cm distal to the tip of the greater trochanter to remove the obstruction of the proximal femoral part. The short section of the vastus lateralis was lifted to access the subtrochanteric area. Then proximal femoral fragment was translated anteriorly to expose the true acetabulum which was further reamed to prepare a socket for acetabular components. The press-fit technique was used in all implantation of the acetabular (Pinnacle, DePuy) with two or three screws to improve primary stability. Structural autograft or titanium alloy (Ti-alloy) mesh was used to increase coverage if necessary. Then the second transverse subtrochanteric osteotomy was conducted as previously planned. The femoral canal was reamed sequentially to get appropriate femoral component size. If reduction with trials could not be achieved, an additional transverse osteotomy was performed to facilitate the hip reduction in the true acetabulum. The straight stem (S-ROM, DePuy) was inserted at 15° to 20° of anteversion in all patients by adjusting the rotational alignment of the 2 fragments. Eventually, C-arm X-ray was used to ensure the position of the femoral and acetabular prosthesis.

Intraoperative cell salvage collection system (3000P, Jingjing Medical Equipment, Beijing, China) was used for all patients. Any fluid visibly contaminated was not salvaged. According to the result of hemoglobin (Hb) and hematocrit (HCT) in intraoperative blood gas analysis, the automatic (medium speed) program was started to process and reinfuse salvaged blood cells with an HCT of 30–40% once estimated blood loss exceeded 500 ml. In consistent with the perioperative transfusion guidelines of the Chinese Ministry of Health, allogeneic RBC transfusion was indicated for a Hb level of < 70 g/L, except for patients with any anemia-related organ dysfunction or intolerable symptoms of anemia [15].

### Assessments

**Fig. 1:**
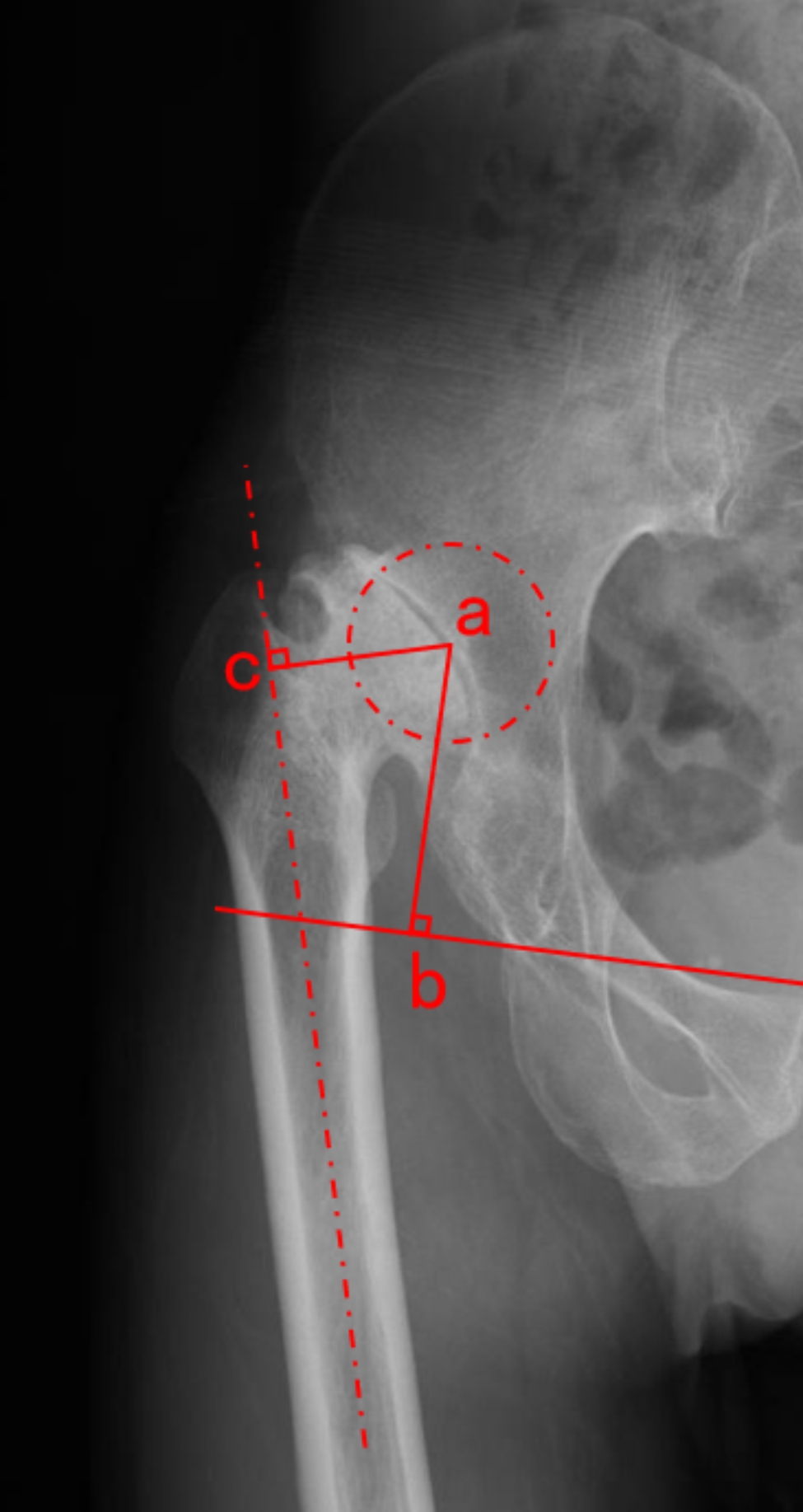
The center of rotation (COR) height and femoral offset (FO) were measured on the AP pelvic radiograph. The COR height (ab): the perpendicular distance between the center of rotation and the interteardrop line. FO (ac): the perpendicular distance between the center of rotation and the axis of the femoral shaft

The clinical and radiographic variables in this study are reported in Table 1. All demographic data were collected including age, sex, height, weight, body mass index (BMI), and tobacco use. Clinical evaluations were conducted preoperatively including the American Society of Anesthesiologists (ASA) classification, Crowe classification, range of hip motion, Harris Hip Score (HHS), and limb-length discrepancy (LLD) [16, 17]. The limb-length discrepancy (LLD) was measured by calculating the difference of distance from the anterior superior iliac spine to the medial malleolus between lower extremities [18]. The radiographic data of X-ray included preoperative femoral offset (FO) and center of rotation (COR) height (Fig. 1). The FO was defined as the perpendicular distance between the center of rotation and the axis of the femoral shaft [19]. The COR height was defined as the perpendicular distance between the center of rotation and the interteardrop line [20]. The intraoperative variables were obtained from patient medical records including the length of SSO, the use of drainage, the duration of operation, the volume of reinfusion from blood salvage, intraoperative complication (intraoperative periprosthetic femoral fracture), and postoperative allogeneic blood transfusion (rates and units) within 3 days of implantation. In addition, the preoperative blood examinations were also recorded including Hb, HCT, platelet (PLT), prothrombin time (PT), and activated partial thromboplastin time (APTT).

**Table 1:** Clinical characteristics of all recruited patients. Values are mean (range) or number

Variable	Reinfused cases (n = 107)	Non-reinfused cases (n = 71)	*p* value
Age, y	46.8 (19–79)	46.4 (18–74)	0.513
Sex			0.841
male	89	58	
female	18	13	
Height, m	1.55 (1.40–1.78)	1.56 (1.40–1.75)	0.178
Weight, kg	55.3 (37–78)	55.4 (37–70)	0.839
BMI, kg/m^2^	23.1 (17.0–31.0)	22.8 (15.4–28.7)	0.641
ASA classification			0.884
I	36	26	
II	49	30	
III	22	15	
Tobacco use	14	16	0.107
Crowe classification			0.317
III	16	15	
IV	91	56	
HHS	43.9 (26–61)	41.3 (20–65)	0.075
ROM, °	117.0 (15–225)	122.9 (30–220)	0.577
LLD, mm	28.4 (0–60.0)	17.1 (0–51.0)	**< 0.001** ^a^
FO, mm	36.1 (20.0–50.0)	35.7 (21.0–48.0)	0.527
COR height, mm	60.3 (28.0-107.0)	44.9 (14.0–81.0)	**< 0.001** ^a^
Preoperative Hb, g/L	132.9 (93.0-176.0)	130.9 (85.0-164.0)	0.586
Preoperative HCT, L/L	0.40 (0.29–0.54)	0.40 (0.27–0.48)	0.636
Preoperative PLT, 10^9/L	183.2 (86.0-437.0)	189.7 (69.0-362.0)	0.511
Preoperative APTT, seconds	27.3 (16.0-35.3)	28.3 (20.2–40.5)	0.056
Preoperative PT, seconds	11.2 (9.8–12.8)	11.3 (9.4–13.6)	0.374
Length of SSO, mm	33.4 (1.0–60.0)	24.2 (5.0–50.0)	**< 0.001** ^a^
Intraoperative periprosthetic femoral fracture	11	6	0.798
Drainage use	35	26	0.630
Duration of operation, min	139.2 (95–180)	132.9 (97–190)	0.171
Reinfused blood, ml	246.1 (150–700)	-	
Allogeneic RBC transfused	18	22	**0.029** ^a^
Allogeneic RBC transfused, units	2.7 (1.0–4.0)	2.5 (1.5-4.0)	0.368
Plasma transfused	5	7	0.225
Plasma transfused, ml	320.0 (200–600)	314.3 (200–800)	0.771

BMI, body mass index; ASA, American Society of Anesthesiologists; HHS, Harris Hip Score; ROM, range of motion; LLD, leg length discrepancy; FO, femoral offset; COR, center of rotation; Hb, hemoglobin; HCT, hematocrit; PLT, platelet; APTT, activated partial thromboplastin time; PT, prothrombin time; SSO, subtrochanteric shortening osteotomy; RBC, red blood cell

^a^ P < 0.05 and the values are marked in bold

### Statistical analysis

All statistical analyses were executed using SPSS v25.0 (IBM, Armonk, NY). Continuous variables were expressed as mean with range and compared using an unpaired 2-tailed t-test or Mann-Whitney U test. Categorical variables were compared using the χ^2^ test or Fisher exact test. Logistic regression analysis was used to evaluate the independent risk factors of postoperative allogeneic RBC transfusions within 3 days of implantation and the ability for reinfusion from ICS. In all logistic regression analyses, continuous variables were categorized by their median to relax the linear relationship assumptions. All variables associated with postoperative allogeneic RBC transfusions and the ability for reinfusion from ICS at a significant level in the univariate logistic regression analysis were candidates for stepwise multivariate analysis. In all analyses, P < 0.05 was considered statistical significance.

## Results

### Patients’ demographics

We initially identified a total of 216 patients who received THA combined with SSO for high hip dislocation. Of these, patients who underwent THA with SSO due to pyogenic arthritis (n = 19), those who had received operative intervention for the purpose of treating DDH (n = 4), those who underwent preoperative anticoagulation therapy (n = 3), those who revived ABT intraoperatively or refused ABT perioperatively (n = 5), and those who did not follow the routine practices of TXA and hypotensive anesthesia in our institution (n = 7) were excluded. The final cohort consisted of 178 patients was included: patients who received reinfusion of ICS (n = 107) and those who receive ICS without sufficient collection for reinfusion (n = 71). To further display the feature of the cohort, we divided the cohort into the reinfusion and non-reinfusion groups (Table 1).

### Allogeneic blood transfusions and independent risk factors

In total, 40/178 (22.5%) patients received postoperative allogeneic RBC transfusion up to 3 days after implantation: 18/107 (16.8%) in the reinfusion group, and 22/71 (31.0%) in the non-reinfusion group. The rate of postoperative allogeneic RBC transfusion was significantly lower in patients who received reinfusion of ICS than in the non-reinfusion group (P = 0.029). The majority of allogeneic RBC (29/40, 72.5%) was transfused on postoperative day 1. There was no difference in the number of patients who received postoperative fresh frozen plasma transfusion between the two groups. In addition, there was also no significant difference between the 2 groups in the volume of postoperative allogeneic RBC and fresh frozen plasma transfusion per case (2.7 units vs. 2.5 units, P = 0.368; 320 ml vs. 314.3 ml, P = 0.771). The intraoperative factors and postoperative results related to ABT are also shown in Table 1. Univariate logistic regression analysis showed that the reinfusion of ICS, BMI ≥ 20.5 kg/m^2^, the COR height ≥ 60 mm, the length of SSO ≥ 30 mm, and the use of drainage were associated with postoperative allogeneic RBC transfusion. The results of univariate analysis of postoperative allogeneic RBC transfusion are shown in Table 2. After adjustment for covariates with the use of the multivariate logistic model, the reinfusion of ICS (OR (95%CI) 0.17 (0.07–0.47)), the COR height ≥ 60 mm (OR (95%CI) 3.30 (1.21–9.01)), the length of SSO (OR (95%CI) 2.75 (1.05–7.22)) and the use of drainage (OR (95%CI) 2.28 (1.04–5.03)) were identified as independent factors associated with postoperative allogeneic RBC transfusion. The results of the multivariate logistic regression analysis are also summarized in Table 2.

**Table 2:** Logistic regression analysis of postoperative allogeneic RBC transfusions in the Cohort

Variable	OR (95% CI)	*P* value
Univariate Logistic Regression Analysis		
Age, ≥ 48 vs. < 48 y	0.85 (0.42–1.73)	0.660
Sex, female vs. male	1.25 (0.48–3.31)	0.648
Height, ≥ 1.55 vs. < 1.55 m	0.57 (0.28–1.16)	0.120
Weight, ≥ 55 vs. < 55 kg	0.74 (0.37–1.49)	0.399
BMI, ≥ 20.5 vs. < 20.5 kg/m^2^	0.45 (0.21–0.98)	**0.044** ^a^
ASA classification		0.223
II vs. I	1.46 (0.66–3.19)	0.349
III vs. I	0.59 (0.19–1.81)	0.356
Tobacco use, Yes vs. No	0.65 (0.23–1.81)	0.406
Crowe classification, III vs. IV	2.19 (0.72–6.68)	0.169
HHS, ≥ 44 vs. < 44	1.17 (0.58–2.37)	0.660
ROM, ≥ 120 vs. < 120 °	0.82 (0.40–1.67)	0.581
LLD, ≥ 25 VS < 25 mm	2.00 (0.98–4.08)	0.057
FO, ≥ 38 VS < 38 mm	0.70 (0.34–1.42)	0.320
COR height, ≥ 60 vs. < 60 mm	2.23 (1.08–4.60)	**0.030** ^a^
Preoperative Hb, ≥ 130 vs. < 130 g/L	0.84 (0.41–1.70)	0.618
Preoperative HCT, ≥ 0.4 vs. < 0.4 L/L	0.81 (0.40–1.65)	0.561
Preoperative PLT, ≥ 160 vs. < 160 10^9/L	1.29 (0.60–2.75)	0.519
Preoperative APTT, ≥ 27.5 vs. < 27.5 s	1.10 (0.54–2.25)	0.786
Preoperative PT, ≥ 11.1 vs. < 11.1 s	1.01 (0.50–2.06)	0.977
Length of SSO, ≥ 30 vs. < 30 mm	2.33 (1.10–4.96)	**0.028** ^a^
Intraoperative periprosthetic femoral fracture	0.491 (0.17–1.42)	0.190
Drainage use, Yes vs. No	2.37 (1.15–4.86)	**0.019** ^a^
Duration of operation, ≥ 150 vs. < 150 min	1.11 (0.53–2.33)	0.777
Reinfusion of ICS, Yes vs. No	0.45 (0.22–0.92)	**0.029** ^a^
Multivariate logistic regression analysis		
BMI, ≥ 20.5 vs. < 20.5 kg/m^2^	0.46 (0.20–1.08)	0.076
COR height, ≥ 60 vs. < 60 mm	3.30 (1.21–9.01)	**0.020** ^a^
Length of SSO, ≥ 30 vs. < 30 mm	2.75 (1.05–7.22)	**0.040** ^a^
Drainage use, Yes vs. No	2.28 (1.04–5.03)	**0.041** ^a^
Reinfusion of ICS, Yes vs. No	0.17 (0.07–0.47)	**< 0.001** ^a^

BMI, body mass index; ASA, American Society of Anesthesiologists; HHS, Harris Hip Score; ROM, range of motion; LLD, leg length discrepancy; FO, femoral offset; COR, center of rotation; Hb, hemoglobin; HCT, hematocrit; PLT, platelet; APTT, activated partial thromboplastin time; PT, prothrombin time; SSO, subtrochanteric shortening osteotomy; RBC, red blood cell

^a^ P < 0.05 and the values are marked in bold

### Salvaging sufficient blood for reinfusion and independent risk factors

Except for the length of SSO which can be estimated before surgery, all variables used in this analysis were based on the data obtained preoperatively. Univariate logistic regression analysis showed that the COR height ≥ 60 mm, LLD ≥ 25 mm, and the length of SSO ≥ 30 mm were associated with the ability to salvage sufficient blood for reinfusion. The results of univariate logistic analysis are presented in Table 3. On multivariate analysis, COR height ≥ 60 mm (OR (95%CI) 3.47 (1.58–7.61)), LLD ≥ 25 mm (OR (95%CI) 2.55 (1.15–5.65)) and the length of SSO ≥ 30 mm (OR (95%CI) 2.75 (1.33–5.69)) were independently associated with the ability for reinfusion from ICS (Table 3).

**Table 3:** Logistic regression analysis of salvaging sufficient blood for reinfusion in the Cohort

Variable	OR (95% CI)	*P* value
Univariate Logistic Regression Analysis		
Age, ≥ 48 vs. < 48 y	1.75 (0.95–3.20)	0.072
Sex, female vs. male	1.11 (0.51–2.43)	0.798
Height, ≥ 1.55 vs. < 1.55 m	0.56 (0.30–1.03)	0.062
Weight, ≥ 55 vs. < 55 kg	0.75 (0.41–1.36)	0.341
BMI, ≥ 20.5 vs. < 20.5 kg/m^2^	1.41 (0.70–2.86)	0.337
ASA classification		0.889
II vs. I	1.18 (0.60–2.33)	0.633
III vs. I	1.06 (0.46–2.42)	0.892
Tobacco use, Yes vs. No	0.52 (0.24–1.14)	0.103
Crowe classification, III vs. IV	1.52 (0.70–3.32)	0.290
HHS, ≥ 44 vs. < 44	1.78 (0.97–3.27)	0.063
ROM, ≥ 120 vs. < 120 °	0.84 (0.46–1.53)	0.560
LLD, ≥ 25 VS < 25 mm	4.38 (2.15–8.92)	**< 0.001** ^a^
FO, ≥ 38 VS < 38 mm	1.47 (0.80–2.69)	0.210
COR height, ≥ 60 vs. < 60 mm	6.78 (3.39–13.57)	**< 0.001** ^a^
Preoperative Hb, ≥ 130 vs. < 130 g/L	1.15 (0.63–2.12)	0.645
Preoperative HCT, ≥ 0.4 vs. < 0.4 L/L	1.20 (0.65–2.21)	0.558
Preoperative PLT, ≥ 160 vs. < 160 10^9/L	1.19 (0.63–2.23)	0.591
Preoperative APTT, ≥ 27.5 vs. < 27.5 s	0.59 (0.32–1.09)	0.091
Preoperative PT, ≥ 11.1 vs. < 11.1 s	0.66 (0.36–1.22)	0.183
Length of SSO, ≥ 30 vs. < 30 mm	4.68 (2.46–8.92)	**< 0.001** ^a^
Multivariate logistic regression analysis		
COR height, ≥ 60 vs. < 60 mm	3.47 (1.58–7.61)	**0.002** ^a^
LLD, ≥ 25 VS < 25 mm	2.55 (1.15–5.65)	**0.021** ^a^
Length of SSO, ≥ 30 vs. < 30 mm	2.75 (1.33–5.69)	**0.007** ^a^

BMI, body mass index; ASA, American Society of Anesthesiologists; HHS, Harris Hip Score; ROM, range of motion; LLD, leg length discrepancy; FO, femoral offset; COR, center of rotation; Hb, hemoglobin; HCT, hematocrit; PLT, platelet; APTT, activated partial thromboplastin time; PT, prothrombin time; SSO, subtrochanteric shortening osteotomy

^a^ P < 0.05 and the values are marked in bold

## Discussion

In our study, the reinfusion of autologous blood from ICS significantly reduced the exposure rate of postoperative allogeneic RBC transfusion within 3 days of implantation in patients who received THA combined with SSO for high hip dislocation. In addition, approximately 40% of patients could not produce sufficient blood salvage volume for reinfusion during THA combined with SSO. Our study suggested that LLD, COR height, and length of SSO were identified as independent factors associated with the ability to salvage sufficient blood for reinfusion.

Due to more restrictive transfusion thresholds, several large randomized controlled trials have indicated that cell salvage could neither reduce the need for allogeneic RBC transfusion nor improve the overall cost-effectiveness in elective total hip and knee arthroplasty procedures [21, 22]. However, there are no published data regarding the role of ICS in THA combined with SSO. With sufficient blood collected for processing and reinfusion, the risk of allogeneic red blood cell transfusion within 3 days of implantation was significantly reduced in this study. Similar clinical outcomes have also been reported in previous reports regarding THA revision; Liu et al. found that ICS could reduce the exposure of postoperative ABT in patients receiving second-stage reimplantation for the treatment of chronic hip periprosthetic joint infection, and Palmer et al. reported that the reinfusion of ICS equated to nearly one unit of packed RBC each patient in revision hip arthroplasty [23, 24]. In this study, we also found that the use of drainage increased the risk of postoperative RBC transfusion in patients who received THA with SSO. Several previous randomized controlled trials reported that closed-suction drainage could eliminate the tamponade effect to increase the rate of homologous blood transfusion which was further confirmed in the present study [25, 26].

Not all patients could be collected adequate blood for the re-transfusion system during THA with SSO. For example, adequate blood for reinfusion was collected in only 40% of patients during the revision hip arthroplasty [27]. Therefore, the importance of appropriate case selection should be emphasized to help allocate cell salvage when there was limited resources. The ability to salvage enough blood for process and reinfusion was largely hinged on the volume of intraoperative blood loss [24]. The decision to use ICS ought to be anticipated preoperatively according to the information available. In the present study, greater COR height was proved to enhance the ability to collect adequate blood for reinfusion in THA combined with SSO. Severe morphologic consequences of great rotation center height could result in extensive soft tissue injury during the surgery and require long operation time, which could produce more intraoperative blood loss for ICS [28]. However, no association was found between the reinfusion from ICS and the Crowe classification. The possible interpretations might include the following: first, the sample size of Crowe type III patients included in this study was still small limiting the analysis and the included patients might not be that representative; second, since the Crowe classification is based on the percentage of dislocation height to pelvic height, this standardization of Crowe classification in terms of the degree of hip dislocation made it unsuitable for individualized assessment of blood loss.

In addition, the longer length of SSO was also found to be an independent factor of the ability for reinfusion in this study. Although the length of SSO was not a preoperative variable, it was supposed to be planned according to the measurement and calculation of X-ray film. A previous study has reported that longer length of SSO was associated with more intraoperative blood loss [29]. The exposure of the femoral shaft with long incision and muscle release around the thigh increased the chance of reinfusion. In addition, the length of SSO was also affected by the soft tissue condition around the hip. Muscle contracture caused by developmental deformities might require longer length of SSO to avoid nerve stretching and to facilitate hip joint reduction [13]. Therefore, to make accurate decision, it was necessary to calculate the length of osteotomy preoperatively according to comprehensive evaluation of patients.

There was no report on the association between LLD and the ability for reinfusion from ICS. Fujimaki et al. reported that the clinical outcomes of patients with postoperative LLD of < 5 mm were superior to those of patients with postoperative LLD of ≥ 5 mm [30]. Therefore, one of the surgical purposes was leg length correction which required extending the affected limb without stretching nerves. In addition, the formation of LLD was complex: Sugano et al. reported that patients with chronic dislocated hips normally had a smaller diameter of the femoral head and shorter femoral neck than normal population [31]; Zhang et al. found that most DDH patients developed LLD due to greater ipsilateral tibial length, skeletal limb length, and lesser trochanter-tibial plafond distance [32]. Thus, to correct skeletal lower limb length, demanding surgical technique was requisite including extensive tissue release of pelvifemoral muscles such as iliopsoas and repeated attempts of hip reduction in the anatomic acetabulum [33]. Those operative procedures could prolong the operation duration and generate sufficient blood salvage volume. One particular advantage of the present study was that it took into account not only radiographic and operative variables but also a wide array of other variables previously reported to be associated with intraoperative blood loss. Greenky et al. reported that elder population produced less amount of salvaged blood [12]; Palmer et al. found that males generated more volumes of autologous blood for reinfusion than females because of greater circulating volumes in males [24]. However, our study suggested that patient factors were not independent determinants of the ability to salvage enough blood for reinfusion. Similarly, preoperative Hb, PLT, and coagulation function were also not associated with the ability to collect sufficient blood for reinfusion. Those results might be explained by the fact that high hip dislocation secondary to DDH was more common in young females which made previously reported factors relatively homogenous [34].

Our study has several strengths. First, we confirmed the effect of ICS on postoperative ABT in patients who underwent THA with SSO for high hip dislocation, which could not only help clinical decision-making but also enable patients to actively cooperate with surgeons by enhancing patients’ cognition. Second, our study also found the association between several risk factors and the ability to generate adequate blood salvage for reinfusion which could optimize the resources and efficiency by targeting patients who are most likely to benefit from ICS during THA combined with SSO. In addition, since the risk factors identified in the present study might influence perioperative blood management, clinicians should emphasize the physical examination and the measurement of X-ray film in this specific situation where severe deformities of lower extremities are usually present [32]. Several limitations existed in the present study. First, this analysis was based on data from single institution. Although we recognized there were numerous differences between patients and institutions, the results of our study could provide surgical teams with an estimation of the reinfusion from ICS for THA with SSO. Second, a prospective study with more fastidious suctioning was required to further confirm the reliability of our outcomes. Third, the sample size was still small which could limit some analyses. Finally, the data on certain factors, such as the deformity of acetabulum and i.v. fluid therapy, was unavailable; therefore, the effect or potential incorporation could not be assessed.

## Conclusion

The use of ICS was an effective blood conservation strategy during THA with SSO for the treatment of high hip dislocation secondary to DDH, which could decrease the exposure rate of postoperative RBC transfusion. After adjusting for other potentially important confounding factors, we demonstrated that the ability to salvage sufficient blood for reinfusion was associated with the COR height, LLD, and length of SSO. Our findings might help clinical decision-making in the context of resource utilization.

## Data Availability

The datasets used and/or analyzed during the current study are available from the corresponding author on reasonable request.

## List of Abbreviation

ICS: intraoperative cell salvage
THA: total hip arthroplasty
SSO: subtrochanteric shortening osteotomy
ABT: allogeneic blood transfusion
RBC: red blood cell
BMI: body mass index
ASA: American Society of Anesthesiologists
HHS: Harris Hip Score
ROM: range of motion
LLD: leg length discrepancy
FO: femoral offset
COR: center of rotation
Hb: hemoglobin
HCT: hematocrit
PLT: platelet
APTT: activated partial thromboplastin time
PT: prothrombin time
SSO: subtrochanteric shortening osteotomy
RBC: red blood cell
